# RETRACTION: Protective Effects of Fruit Extract of *Rosa canina* and Quercetin on Human Umbilical Vein Endothelial Cell Injury Induced by Hydrogen Peroxide

**DOI:** 10.1002/fsn3.70126

**Published:** 2025-04-09

## Abstract

**RETRACTION**: F. Forouzanfar, Z. Tabatabaei, S. A. Emami, Z. Ayati and Z. Tayarani‐Najaran, “Protective Effects of Fruit Extract of *Rosa canina* and Quercetin on Human Umbilical Vein Endothelial Cell Injury Induced by Hydrogen Peroxide,” *Food Science & Nutrition* 11, no. 12 (2023): 7618–7625, https://doi.org/10.1002/fsn3.3681.

The above article, published online on 28 September 2023 in Wiley Online Library (wileyonlinelibrary.com), has been retracted by agreement between the journal Editor‐in‐Chief, Y. Martin Lo; and Wiley Periodicals LLC. The retraction has been agreed upon as some of the western blot bands shown in Figure 4 are inconsistent with the corresponding quantification graphs. Furthermore, the use of the ANOVA parametric test on *n* = 3 biological replicates is inappropriate, leading to unreliable statistical analysis. The authors provided an explanation and some raw data, but this was deemed insufficient to resolve the flaws in the published manuscript. The editors consider the results in this article unreliable. The authors disagree with the retraction.

**RETRACTION**: F. Forouzanfar, Z. Tabatabaei, S. A. Emami, Z. Ayati and Z. Tayarani‐Najaran, “Protective Effects of Fruit Extract of *Rosa canina* and Quercetin on Human Umbilical Vein Endothelial Cell Injury Induced by Hydrogen Peroxide,” *Food Science & Nutrition* 11, no. 12 (2023): 7618–7625, https://doi.org/10.1002/fsn3.3681.

The above article, published online on 28 September 2023 in Wiley Online Library (wileyonlinelibrary.com), has been retracted by agreement between the journal Editor‐in‐Chief, Y. Martin Lo; and Wiley Periodicals LLC. The retraction has been agreed upon as some of the western blot bands shown in Figure 4 are inconsistent with the corresponding quantification graphs. Furthermore, the use of the ANOVA parametric test on *n* = 3 biological replicates is inappropriate, leading to unreliable statistical analysis. The authors provided an explanation and some raw data, but this was deemed insufficient to resolve the flaws in the published manuscript. The editors consider the results in this article unreliable. The authors disagree with the retraction.

